# Hydrophobia

**Published:** 1889-06-29

**Authors:** 


### HYDROPHOBIA.

*(Concluded from page 146.)*

Notwithstanding that scientists, both in British and continental, after experiment, fully supported M. Pasteur's views, it was not long before a reaction set in; for various maladies as well as hydrophobia, and even death, were asserted to have followed Pasteur's treatment. The editor of the *Journal de Medicine* said, "M. Pasteur does not cure hydrophobia; he gives it." In a lecture by M. Lutard at Prince's Hall, Piccadilly, July, 1887, it was contended that the system had increased instead of diminishing hydrophobia mortality. Unfavourable results after Pasteur's treatment have been noted in the *British Medical Journal* for August 27, September 3, October 8, 1887; in the *Lancet* of November 13, October 23, 1886, June, 1887, November 3, 1888, the *Provincial Medical Journal*, June 1, 1889, and in other places. Against this, however, it has been asserted that out of 233 bitten by rabid dogs and inoculated only four died, while without treatment forty would have probably died. Every case treated by M. Pasteur for some time since has been placed in one of three classes: A. Where the fact of rabies has been determined by experimental inoculation. B. Those provided with a certificate of the animal inflicting the injury being rabid. C. Those where rabies is merely affirmed on general report. It is stated that in the first class the mortality after Pasteur's treatment has been 1·36 per cent., whereas had the treatment not been followed it would have been 15 per cent. A committee of medical authorities appointed in 1887 to examine into the question were of opinion that "deaths have occurred under conditions which have suggested that they were due to the inoculation rather than the infection." Nevertheless, the committee opined from the evidence before them, that the inoculations practised by M. Pasteur have prevented the occurrence of hydrophobia in a large proportion of those who, if they had not been so inoculated, would have died of the disease. But the committee did not follow the advice of certain scientists, and of certain medical journals, and recommend that a Pasteur institute for the treatment of rabid dogbite should be established in England. Neither did the Lords Committee do so, whose report was issued in August, 1887. This committee, without entering on the scientific question, based their position on the fact that rabies is comparatively rare even in dogs, and that a small proportion of the persons bitten are liable to hydrophobia. It is also worthy of note that the Milanese Commission, in 1888, required further proof of the efficiency of the Pasteur method before advising their Government to accept it. Perhaps the manner in which Dr. Gowers sums up the matter in his recent work on "Nervous Diseases," best meets the position. Dr. Gowers says there are two facts patent—viz., that Pasteur's treatment produced rabies in one case, and that rabies has developed afterwards.

So far back as the year 1844, Dr. John Wright, Physician to the Queen's Hospital, Birmingham, demonstrated that healthy saliva, when injected into the veins, produces symptoms closely resembling, if not identical with, those of hydrophobia. This is a matter which our scientists might well reinvestigate, for it may have an important bearing on various points connected with hydrophobia and its treatment. If Wright's views are correct, and saliva of any kind will excite symptoms of hydrophobia, we have an explanation of so-called false hydrophobia. It would be well to know if healthy saliva will produce rabbit rabies.

It must be recollected that most of the cases sent to Pasteur have been previously cauterised or otherwise treated, and cauterisation may have had a greater influence on the favourable result than Pasteur admits. It is known that rabies poison, unlike snake venom, does not produce immediate effects. It may be months or even years before symptoms of hydrophobia develop. It is tolerably certain that rabies venom remains in or near the wound, and does not, like snake venom, invade the system at once. It is therefore reasonable to suppose that rabies venom may be destroyed if some means are adopted immediately. Mr. Youatt, who wrote a good deal on the subject, stated he had been bitten by mad dogs four times himself, but having used nitrate of silver freely to the wounds he did not experience any after effects. Mr. Youatt is also said to have similarly operated on some 400 persons, all bitten by dogs, respecting the nature of whose disease there could be no question, and not a single case was lost. Others, however, think that nitrate of silver is not a sufficiently powerful caustic, and, moreover, object that when in contact with the tissues it is converted into inert chloride of silver. On the other hand, it has been asserted (*British Medical Journal*, Jan., 1886), that "the preventive benefit of early cauterisation" (by some means) "is as much a fact as hydrophobia itself." Personally we would rather trust to sucking of the part in the first instance, and to nitric acid, carbolic acid, or acetic acid, or the actual cautery in the shape of a heated wire afterwards. It has been stated that sucking the wound may be dangerous, but this is not the fact, unless there is a sore in the mouth or on the lips of the person performing the office. Sucking the wound is as old at least as the days of Celsus, and has been and is practised now in various remote districts and among semi-civilised people. There have been, indeed, persons who made it an occupation. Dr. Maccall (in the *Lancet*, December 24, 1887) suggested prepared points of compressed nitrate of potash ten parts, and corrosive sublimate two parts, which might be kept in a case attached to the key-ring, and used by the police in case of dogbite. Points of permanganate of potash have also been recommended for similar use. It has certainly appeared that treatment of the wound, whether by caustics or excision, does sometimes prevent hydrophobia, and the not unreasonable question has been asked, when a person's wound has been so treated and the person afterwards subjected to Pasteur's treatment, and does not get hydrophobia, Which was the means of prevention?

There is another matter which must also be taken into consideration—viz., the incubation of hydrophobia. M. Pasteur is of opinion that symptoms of hydrophobia usually show within the first five months after a person is injured by a rabid animal. But a case has been mentioned occurring four days after a bite. A recent authority (Dolan) thinks that the shortest period on reliable record is twelve days, and that it is rarely less than a month. On the other hand, cases have been recorded when hydrophobia did not appear until three, or even twelve years had passed. In the *British Medical Journal* for February 28, 1881, cases are recorded of upwards of twenty months, and of upwards of ten months incubation. A case recently occurred at Dalhousie, India, of thirteen months. In the *Indian Medical Gazette* for 1887 a case of one and a half years' incubation is reported. In the *Transactions* of the Bombay Medical and Physical Society for 1860-61 an instance is given of three years' delay. A remarkable case has been recorded of a man who had been in prison for more than three years becoming affected. As the poison of syphilus may remain in the system without producing recognisable results for an unlimited period, and as malarious poison may do the same, there is no valid reason why rabies poison should not also remain latent. It has been truly observed that it is quite as difficult to explain an incubation of six months as of six years. Now, before a cure by any means can be asserted, it is necessary that this question of incubation should be cleared up. There is no doubt that a case of hydrophobia has occurred three years after an injury, whatever scepticism may be felt as to twelve years or longer periods. Neither Pasteur's nor any other treatment can therefore be regarded as successful until at least three years have passed away.

After the reports of the committees previously referred to, as presented in 1887, Government, as we think, wisely declined to sanction the establishment at the public expense of a Pasteur institute in this country for the treatment of hydrophobia. In these days of onward scientific progress it is well not to deny anything, and it is quite possible that Pasteur's treatment may eventually be established beyond all doubt. At present we agree with the Milanese Commission that further proof is required, and to this we add also that more time is required, for, as previously mentioned, the incubation of hydrophobia may be years. A resumé of the reasons which induced Government to decline acceding to the pressure brought to bear on them will show that Government were correct. 1. Many cases reported as hydrophobia are not so, being pseudo, mental, or false hydrophobia. 2. Various maladies have been mistaken for hydrophobia. 3. Numbers bitten do not suffer from any after effects. 4. Hydrophobia is not a necessarily fatal disease. 5. Hydrophobia is, comparatively speaking, not a prevalent malady. 6. Hydrophobia is not more terrible than certain other diseases. 7. Pasteur's system is not always successful, and has been followed by unfavourable results. 8. The incubation of hydrophobia not being known, it cannot be asserted that time has elapsed to secure Pasteur's treatment successful results. 9. The question is not satisfactorily answered, whether early treatment of the wound, or subsequent treatment by Pasteur, has caused immunity, so far. 10. Police reguations may be enforced sufficient to prevent hydrophobia.

Youatt long since maintained that if every dog in the United Kingdom were confined separately for seven months, rabies and hydrophobia might be extirpated. This, perhaps, is impracticable, but there are certain police regulations which should always be in force. Wandering dogs should be destroyed. The keeping of useless dogs should be repressed by a prohibition tax. Muzzles of proper character should be used whenever a rabid dog appears in the neighbourhood, and for a specified time afterwards. The importation of dogs should be discouraged by a high duty, and each dog imported should be subject to expert examination. Caustic sticks, as previously mentioned, should be kept at every police-station, and several men should be fully instructed in the use of such antidotes. The symptoms of rabies, which are now printed on a dog license, should also be plainly set up in every police-station. The failure to notify a case of rabies or hydrophobia should be made a penal offence. Dogs suspected of being rabid, and also dogs bitten by suspected dogs, should be carefully confined at the owner's expense, and, in consideration of the long period of incubation, for at least six months. It may, in fact, be stated that rabies is mainly attributable to the absence of suitable sanitary regulations. By appropriate measures rabies may be easily confined within a small area, and probably eventually suppressed. A draft Act was published in 1879 by Dr. Dolan, which appears to meet all wants.

In conclusion, we notice another fallacy connected with the subject. Many persons think that when a dog bites, the individual injured is in danger of hydrophobia if the dog gets rabies afterwards, or even if the dog is bitten by a mad dog. This is quite incorrect, and the practice, founded on the above impression, of killing a dog if it bites anyone is cruel and unnecessary. It is advisable that the dog should live, and certainty attained that the animal was not rabid. The idea mentioned above is as ridiculous as, and possibly a relic of, the belief mentioned in Thomas Lupton's "Notable Things" (1660): "Whosoever is stricken or hurt by any venomous worm or other thing, or else bitten by a mad dog, let them take heed diligently that the same thing that did hurt them see them not until they be whole. For the Hebrew physitians say that the party hurt shall then die."

